# Alterations of Fucosyltransferase Genes and Fucosylated Glycans in Gastric Epithelial Cells Infected with *Helicobacter pylori*

**DOI:** 10.3390/pathogens10020168

**Published:** 2021-02-04

**Authors:** Ruyue Fan, Xiurui Han, Yanan Gong, Lihua He, Zhijing Xue, Yaming Yang, Lu Sun, Dongjie Fan, Yuanhai You, Fanliang Meng, Xiaomei Yan, Maojun Zhang, Jianzhong Zhang

**Affiliations:** State Key Laboratory of Infectious Disease Prevention and Control, Collaborative Innovation Center for Diagnosis and Treatment of Infectious Diseases, National Institute for Communicable Disease Control and Prevention, Chinese Center for Disease Control and Prevention, Beijing 102206, China; fryforever@163.com (R.F.); hanxiurui211@163.com (X.H.); gongyanan@icdc.cn (Y.G.); helihua@icdc.cn (L.H.); 15069725531@163.com (Z.X.); yangyaming@njmu.edu.cn (Y.Y.); sunlu@icdc.cn (L.S.); fandongjie@icdc.cn (D.F.); youyuanhai@icdc.cn (Y.Y.); mengfanliang@icdc.cn (F.M.); yanxiaomei@icdc.cn (X.Y.); zhangmaojun@icdc.cn (M.Z.)

**Keywords:** *H. pylori*, fucosyltransferase expression, fucosylation, gastric epithelial cells

## Abstract

*Helicobacter pylori* (*H. pylori*) adhesion to human gastric epithelial cells is closely linked with fucosylated glycans. Therefore, investigation of fucosylation in the interaction of gastric epithelial cells with *H. pylori* is critical. In this study we used lectin microarrays to detect the expression of fucosylated glycans in gastric epithelial cells (GES-1) infected with *H. pylori* strains isolated from patients with different diseases including chronic gastritis, duodenal ulcers, and gastric cancer (each containing two strains) at 4 h. In addition, we investigated the time-course expression of fucosyltransferase (FUT) 1–6 genes in GES-1 cells stimulated with *H. pylori* strains at 0.5–8 h. At 4 h post-infection, Lotus, AAA, BC2LCN, PA-IIL, CNL and ACG lectins had increased signals in *H. pylori*-infected GES-1 cells compared to uninfected cells. Higher expression of FUT1 and FUT2 was detected in all *H. pylori*-infected GES-1 cells within 2 h, regardless of the *H. pylori* strain. In particular, the expression of FUT2 was higher in *H. pylori*-infected GES-1 cells with a higher fold change in levels of BC2LCN lectin specific to α1-2 linked fucose (Fuc) at 4 h. The results suggest that the high levels of α1, 2-linked Fuc synthesized by FUT1/2, might play a role in the preliminary stage of *H. pylori* infection. This provides us with pivotal information to understand the adhesion of *H. pylori* to human gastric epithelial cells.

## 1. Introduction

**Figure 1:**
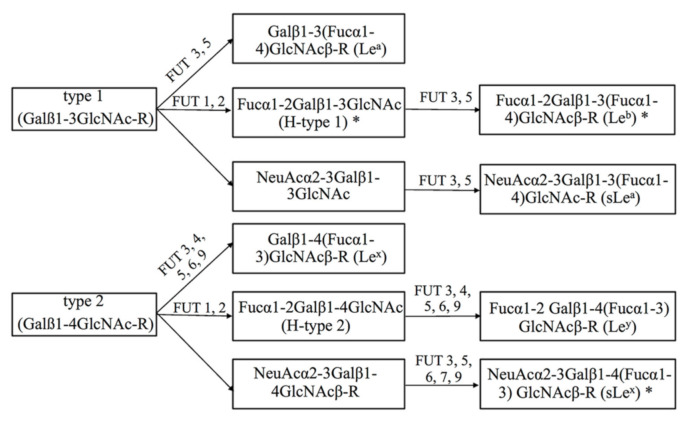
Biosynthetic pathway of the Lewis histo-blood group antigens. Type 1 (H type 1, Le^a^, Le^b^ and sLe^a^) and type 2 (H type 2, Le^x^, Le^y^, and sLe^x^) Lewis antigens are terminal fucosylated carbohydrates in cell surface glycoproteins or glycolipids and are synthesized by fucosyltransferases (FUTs 1–7 and FUT9). FUT1 and FUT2 are α-1,2-fucosyltransferases. FUTs 3–7 together with FUT9 added Fuc in α1-3 linkage. FUT3 and FUT5 have α-1,4-fucosyltransferase activity. *H. pylori* adhesion to gastric epithelial cells is mediated by adhesin BabA binding Lewis b and type 1 structures, while SabA binds sLe^x^ (marked in *).

Fucosylation is one of the most common forms of glycosylation in mammalian cells, participating in various biological processes such as cell–cell communication, host–pathogen interactions and immune responses [1,2]. Importantly, alterations of fucosylated glycans in cell glycoproteins and glycolipids serve as biomarkers of disease progression and indicate the onset of certain diseases [2,3,4,5,6]. Type 1 and type 2 Lewis histo-blood antigens are terminal fucosylated carbohydrates that frequently occur on the surface of cancer cells and are correlated with the progression of cancers [3]. The expression of fucosylated epitopes is mainly attributed to the regulation of pertinent fucosyltransferases (FUTs) encoded by FUT genes [1]. Among the FUTs discovered, several key enzymes catalyze the formation of Lewis blood group antigens, as shown in Figure 1. FUT1 and FUT2 are responsible for the α1-2 linkage of Fuc, forming the H type epitope [7]. FUTs 3–7, together with FUT9, have α-1,3-fucosyltransferase activities and are involved in the formation of Lewis blood group antigens, including Lewis x (Le^x^), sialyl Lewis x (sLe^x^) and Lewis y (Le^y^) [4,7,8]. FUT3 and FUT5 have also been shown to be responsible for the α1-4 linkage of Fuc, resulting in the synthesis of type 1 Lewis antigens, such as Lewis a (Le^a^), Lewis b (Le^b^), and sialyl Lewis a (sLe^a^) [8]. Additionally, sialic acids (Sias) attach to fucosylated glycans via 2–3 or 2–6 linkages to synthesize sialyl fucosylated determinants, which are critical for recognition by pathogens [9]. 

*H. pylori* infection is accepted as the main cause of diseases such as gastritis, duodenal ulcer disease, gastric ulcer disease, gastric adenocarcinoma and gastric mucosa-associated lymphoid tissue (MALT) lymphoma in the world’s population [10,11]. To exert its effects, *H. pylori* mainly resides in the gastric mucosa and mucus layer [12], and the interplay between host and bacteria is closely linked with fucosylated glycans [13,14]. *H. pylori* binds to gastric epithelial cells and extracellular mucus through adhesins. The binding of fucosylated Le^b^ and H-type 1 antigens to adhesin BabA is thought to be important in the initial stage of *H. pylori* infection [15,16,17]. Upon the resulting infection and inflammation, adhesin SabA mediates the binding of the bacterium to the sLe^x^ antigen, resulting in chronic infection [18,19]. Moreover, FUT2-driven fucosylation promotes BabA-mediated *H. pylori* adhesion, which has been connected with pathogenicity [20]. Another study reported that the expression of FUT1 was significantly increased in HT29-MTX-E12 cells infected by *H. pylori* [12].

Recently, lectin microarrays have been used as a primary method to capture the glycosylation of samples [21,22,23,24]. Lectin microarrays consist of a series of lectins immobilized onto a glass slide in the form of high density spots [25]. The lectins can specifically bind to carbohydrate and be labeled by different molecules such as fluorescein, biotin and avidin for fluorescence and electrochemical analysis [26]. According to previous reports, some lectins have been used to study altered glycan structures in gastric cancer samples [15,27]. The advanced techniques could improve the quality control for the differential analysis of mammalian glycans and simultaneously be used to examine subtle differences in glycosylation [25]. Meanwhile, the advent of accurate, sensitive and rapid quantitative reverse transcription PCR (qRT-PCR) has facilitated the study of the expression of host cellular genes and replaced older and time-consuming techniques [28,29].

A rhesus macaque model of *H. pylori* infection showed a huge change in gastric epithelial cells within the first few hours of infection [30]. In the present study, we analyzed the changes of Fuc-related glycans in GES-1 cells infected by *H. pylori* strains from different sources (patients with chronic gastritis, duodenal ulcer disease, or gastric cancer) using the lectins microarray. In addition, we examined the time-varying trend of mRNA levels of FUTs 1–6 by qRT-PCR. Our results yielded valuable information about the effects of *H. pylori* infection on the fucosylation of gastric epithelial cells in the preliminary stage of infection, which is crucial in understand the mechanisms of *H. pylori* adhesion and colonization.

## 2. Materials and Methods

### 2.1. Bacterial Strains and Culture Conditions

A total of six *H. pylori* strains were used in this study. The strains were cultured on a Karmali agar plate supplemented with Karmali Agar base (Oxoid, Basingstoke, Hampshire, UK) containing 15% defibrinated sheep blood and the plate was incubated at 37 °C under microaerobic conditions (5% O_2_, 10% CO_2_ and 85% N_2_) for 72 h. The strains included five clinical isolates from patients with chronic gastritis (YN4-62), duodenal ulcer disease (M84, P164) and gastric cancer (HLJ011, HLJ030), and a *H. pylori* type strain ATCC 43504 isolated from a gastritis patient in Australia [31]. The obtained colony was confirmed by urease, oxidase and catalase traits and inspection of bacterial morphology. All strains were stored at −80 °C in the laboratory until use. 

### 2.2. Cell Lines and Cell Culture

Human gastric epithelial cells (GES-1) were obtained from the laboratory of the Peking University Health Science Center. The cell line was grown in RPMI-1640 medium (Thermo Fisher Scientific, Waltham, MA, USA) supplemented with 10% fetal bovine serum (FBS, Thermo Fisher Scientific, Waltham, MA, USA) at 37 °C and 5% CO_2_ in an incubator.

### 2.3. Infection Procedures

The GES-1 cell line was seeded into 12-well cell plates at a density of 1.5 × 10^5^ cells/well. *H. pylori* strains and GES-1 cells were washed in sterile phosphate-buffered saline (PBS) prior to infection. Bacteria were added to FBS-free RPMI-1640 medium and incubated with cells for 0.5–8 h at a multiplicity of infection (MOI) of 100. The relative number of *H. pylori* was examined using Desicheck (BioMérieux, Marcy-l’Étoile, France). The optical density (OD) at 600 nm was set to count colony forming units of *H. pylori* (1 OD_600_ = 2.2 × 10^8^ colony forming units/mL). After infection, unbound bacteria were washed away using sterile PBS three times. The well containing noninfected GES-1 cells were simultaneously collected as controls. At least two independent experiments were performed for each infection procedure.

### 2.4. RNA Extraction and Quantitative RT-PCR

Total RNA of *H. pylori*-infected cells and the controls was extracted with TRIzol reagent (Life Technologies Corporation, Carlsbad, CA, USA). Reverse transcription and qRT-PCR were performed using probe a one-Step qRT-PCR superMix kit (Transgen Biotech, Beijing, China). Data collection was performed using the ABI QuantStudio6 system according to the manufacturer’s instructions. Gene expression levels were normalized to *GAPDH* and quantified in triplicate. The primers and probes used for the investigated genes were previously described [32,33] and are listed in Table 1. Relative fold changes in gene expression were determined using the threshold cycle (2^–ΔΔCt^) method [33].

### 2.5. Cellular Lysis

After 4 h, infected cells were harvested with the cell lysis buffer (RayBiotech, Norcross, GA, USA), and then cell solutions were incubated on ice for 30 min and mixed. The solution was centrifuged at 13,000 × g for 20 min at 4 °C, and then, the supernatant obtained was used as the cell and strain lysate, followed by lectin microarray experiments. The protein concentrations of samples were quantified by BCA assay (Cwbiotech, Beijing, China).

### 2.6. Lectin Microarrays and Data Analysis

The lectin microarray was produced using 10 lectins specific to Fuc-related glycans (purchased from RayBiotech, Norcross, GA, USA). Microarray detection was performed according to a previous report, with some changes [23]. In brief, 100 μg cell lysates were added into dialysis buffer (1× PBS, pH = 8.0) overnight, and the protein concentration after dialysis by BCA assay was quantified. Then, after dialysis, 30 μg of each protein type was labeled with biotin using labeling reagent (RayBiotech, Norcross, GA, USA), and the labeled product was dialyzed using dialysis buffer for the lectin microarray. The lectin microarray used in the study was set at room temperature for 1 h, dried for 2 h at 37 °C and blocked with sample diluent (RayBiotech, Norcross, GA, USA) for 30 min at room temperature. Labeled samples after dialysis were diluted to 100 μL using sample diluent and were then volume-equivalently loaded onto each block of the array and incubated overnight at 4 °C. The array was then washed with wash buffer (RayBiotech, Norcross, GA, USA). Cy3-streptavidin reagent (RayBiotech, Norcross, GA, USA), diluted with sample diluent, was applied to each block for 1 h at room temperature. A final washing step was performed using wash buffer and the slides were dried by centrifugation at 1000 rpm for 3 min. The microarray was canned using an Agilent SureScan Dx Microarray scanner (Agilent Technologies, Santa Clara, CA, USA).

The microarray image was analyzed at 532 nm for Cy3 detection by GenePix Pro 6.0 software (Axon Instruments Inc., Union City, CA, USA). The median value of each point on the blocks was selected to form a new dataset. The average background was subtracted and the mean value of two replicate points of each sample was calculated for further analysis. The normalized data of the cells infected by *H. pylori* strains were compared with uninfected cells based upon fold changes according to the following criteria: fold changes >1.2-fold or <0.8-fold in the pairs indicated increase or decrease in the fluorescent signal of lectins.

### 2.7. Statistical Analyses

All analyses were conducted using SPSS version 20.0 software (IBM Corp., Armonk, NY, USA) and GraphPad Prism 5 (GraphPad, La Jolla, CA, USA). Statistical differences between samples were calculated using Student’s t test. A two-sided *P* value of 0.05 was considered to be statistically significant.

## 3. Results

### 3.1. Glycopatterns Changed after Interaction of Gastric Epithelial Cells with H. pylori Strains

Using lectin microarrays, nine lectins were altered after the interaction of GES-1 cells with *H. pylori* strains at 4 h, in comparison to uninfected cells (Table 2). Among these lectins, the expression level of Fuc-related glycans recognized by AAA, ACG, BC2LCN, CNL, Lotus, PA-IIL and UEA-I was unchanged or up-regulated (>1.2-fold) in GES-1 cells infected with each *H. pylori* strain detected. In addition, αFuc binder RS-Fuc, Fucα1-6GlcNAc or Fucα1-3LacNAc binder AAL and Galα1-3Galβ1-4GlcNAc or Galα1-3 (Fucα1-2) Gal binder MOA had increased signals in GES-1 cells infected with *H. pylori* strains, except for ATCC 43504.

### 3.2. Glycopatterns in GES-1 Cells Infected by H. pylori Isolates from Different Sources

According to the statistics, the Fuc-related glycopatterns detected in GES-1 cells infected with *H. pylori* strains isolated from gastritis chronic gastritis, duodenal ulcer disease and gastric cancer patients were similar (*p* > 0.1).

### 3.3. Time Course of Relative Expression of FUTs 

**Figure 2:**
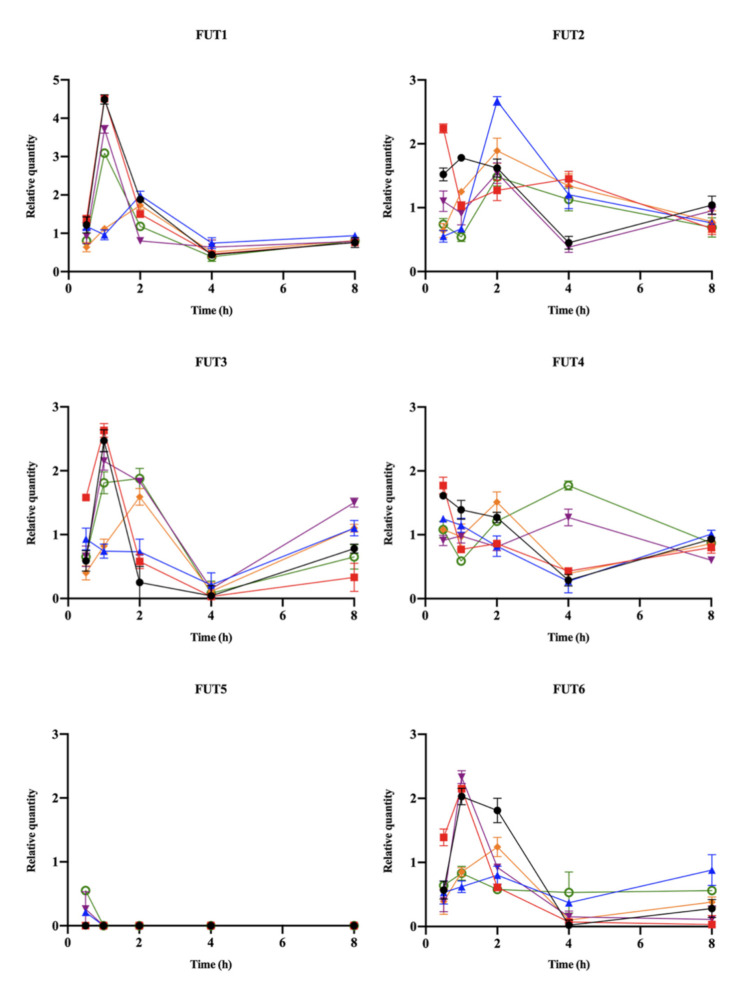
The time-course relative expression of FUT genes in GES-1 cells infected by *H. pylori* isolates. Total RNA from uninfected GES-1 cells and infected GES-1 cells by *H. pylori* isolates ATCC 43504 (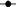), P164 (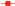), HLJ011 (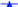), HLJ030 (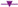), M84 (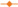), and YN4-62 (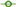) at 0.5–8 h was analyzed using real-time PCR. The expression quantity of FUT genes in uninfected cells was assigned to 1, and the expression level of FUT genes was considered up-regulated in infected cells when the relative quantity > 1.

The relative expression levels of FUTs in GES-1 cells infected with *H. pylori* strains compared to uninfected cells calculated from the actual Ct values are shown in Figure 2. The higher expression of FUT1, FUT2, FUT3, FUT4 or FUT6 in GES-1 infected with *H. pylori* strain ATCC 43504, P164 or HLJ011 could be detected at 0.5 h. The higher expression of FUT1 and FUT2 was detected within 2 h post-infection. The expression of FUT3 in GES-1 cells infected with *H. pylori* strains (except *H. pylori* strain HLJ011) was higher than in uninfected cells within 2 h. At 4 h post-infection, the expression level of FUT2 was higher in GES-1 cells infected with *H. pylori* strain M84, P164, YN4-62, and the expression level of FUT4 was higher in GES-1 cells infected with *H. pylori* strain HLJ030 and YN4-62. The expression of FUT1, FUT3 and FUT6 in GES-1 cells infected with *H. pylori* strains declined over time with only low levels detectable at 4 h, but slightly increased at 8 h. The lower expression levels of FUT5 were only detected in GES-1 cells infected with *H. pylori* strain HLJ011 and YN4-62 at 0.5 h post-infection.

## 4. Discussion

Due to the important roles of fucosylated oligosaccharides—especially fucosylated histo-blood group antigens—in pathogen–cell interactions, such as the *H. pylori* colonization of gastric epithelial cells, the multifactorial analysis of fucosylated glycans and the expression of fucosyltransferases becomes significant [1,23,34]. However, the literature remains limited concerning the changes of fucosylated glyco-epitopes in gastric epithelial cells infected with *H. pylori* strains. In this study, RS-Fuc, Lotus and PA-IIL lectins specific to Fuc showed elevations in GES-1 cell samples infected with five clinical *H. pylori* isolates from different sources at 4 h, indicating Fuc might play a role in the interaction between epithelial cells and *H. pylori* isolates. The difference in results for lectins specific to the same glycan in the microarray might be associated with the origin and characteristics of the lectins, such as the affinity between cells and the carbohydrate structure [35]. Previous studies have reported that di-fucosylated glycans found on the Le^b^ blood group antigen and mono-fucosylated glycans found on the H-type 1 antigen, A antigen, and B antigen of blood group antigens on gastric epithelial cells, were receptors for *H. pylori* adhesin BabA [36,37,38]. The increase in binding of BC2LCN lectin to Fucα1-2Galβ1-3GalNAc and Fucα1-2Galβ1-3GlcNAc (H-type 1 determinant) was found in our study. In addition, we found that clitocybe nebularis lectin—a lectin that specifically binds β1-4GlcNAc and human blood group A determinant-containing glycan epitopes, GalNAcα1-3(Fucα1-2)Galβ1-4GlcNAc and Marasmius oreades lectin, specific to xenotransplantation epitope Galα1-3Galβ1-4GlcNAc and blood group B determinant Galα1-3(Fucα1-2)Gal [39,40]—had increased signals in GES-1 cells infected with *H. pylori* isolates. These results indicated that the α1-2 linkage of Fuc in gastric epithelial cells might be altered after *H. pylori* strain stimulation. A glycoprofiling investigation of gastritis and gastric cancer tissues showed that the expression level of α1-2 linked Fuc was present in high amounts in adenocarcinoma and comparatively lower amounts in gastritis samples [22].

Alterations of fucosyltransferases can modify the glycan expression pattern of the cells [41]. These α1-2 fucosyltransferases (FUT1 and FUT2) are Golgi stack membrane enzymes responsible for the transfer of α1, 2-linked fucose to the galactose (Gal) residue of glycans such as ABH and Lewis blood group antigens [1]. A recent study showed that FUT1/2 induced angiogenesis by activating ERK1/2, accelerated hepatocellular carcinoma progression by influencing Notch signaling, and multidrug resistance by inducing the PI3K/Akt signaling pathway [42]. Our results show that the relative expression of FUT1 and FUT2 was significantly up-regulated in GES-1 cells within 2 h of *H. pylori* strains infection. The highest expression of FUT1 and FUT2 and higher fold change of BC2LCN and MOA lectins in GES-1 cells infected with *H. pylori* strain HLJ011 were also found at 2 h. Specially, FUT2 was higher in GES-1 cells infected with *H. pylori* strains M84, P164, YN4-64 and HLJ011, with a higher fold change in the lectin microarray covering various Fuc patterns, including α1, 2-linked Fuc at 4 h, indicating that FUT2 might play a critical role in the preliminary stage of *H. pylori* infection. It has been reported that the inactivation and mutation of human FUT2 could confer reduced susceptibility to *H. pylori* infection, and the BabA-dependent adhesion of *H. pylori* strains was impaired in the FUT2-null mice [43]. In addition, the dysregulation of FUT2 was related to chronic inflammatory diseases [44]. The changes in expression of FUT1 and FUT2 observed in epithelial cells infected with *H. pylori* strains in this study confer beneficial effects on the adhesion and pathogenicity of *H. pylori* in the stomach.

The expression level of α1-3/6 linked Fuc recognized by AAL and AAA lectins was up-regulated in GES-1 cells infected with *H. pylori* strains, except *H. pylori* ATCC43504, indicating an increase in α1-3/6 linked Fuc in infected epithelial cells appeared within 4 h. The difference in lectins detected in GES-1 cells infected with *H. pylori* ATCC 43504 and other clinical isolates might be related to the sources of strains, reflecting that the characteristics of different strains could result in various glycan alterations induced in host cells. Previous studies have indicated that the interaction between sLe^x^ (NeuAcα2-3Galβ1-4(Fucα1-3) GlcNAcβ-R) and *H. pylori* adhesin SabA could determine the density of bacterial colonization in patients with weak-Leb. When host inflammation increases, expression of sLe^x^ increases, and *H. pylori* strains adhere to the gastric mucosa with adhesin SabA [45]. In our study, the expression of α2-3 Sia, recognized by ACG and Fucα1-6GlcNAc or Fucα1-3GlcNAc recognized by AAA, was increased in the interaction between GES-1 cells and *H. pylori* strains, indicating that the *H. pylori* strains might induce the increase in Le^x^ and sLe^x^. A previous study proved that β3GnT5 transferase overexpression in human gastric carcinoma cell lines caused increased sLe^x^ expression and *H. pylori* adhesion that was mediated by SabA [19]. 

In our study, we evaluated the expression levels of FUTs 3–6 in GES-1 cells infected with *H. pylori* strains over time. FUTs 3–7, together with FUT9 as α-1,3-fucosyltransferase together were responsible for the addition of a Fuc to the N-acetylglucosamine (GlcNAc) of the antenna in α1-3 linkage [1]. In the normal gastric mucosa, cells of the foveolar epithelium express FUT2 and type 1 Lewis antigens (Le^b^, Le^a^ and sLe^a^), deep gland cells express FUT1 and type 2 Lewis antigens (Le^y^, Le^x^ and sLe^x^), and FUT3 is detected in both populations [46]. We found differences in the expression of FUTs 3–6 detected in GES-1 cells infected with various *H. pylori* strains. It should be noted that the higher relative expression of FUT4 was found in all infected GES-1 cells at 0.5 h, showing that the FUT might change in normal gastric epithelial cells infected with *H. pylori* at earlier time points. FUT3 and FUT5 have also been shown to be responsible for the α1-4 linkage of fucose, and we only detected the low expression of FUT5 in GES-1 cells by *H. pylori* strains YN4-62, HLJ011 and HLJ030 infection at 0.5 h. As a previous study described, FUT5 was absent or weakly expressed under normal stomach conditions, whereas levels were increased in gastric tumors [41,47]. The previous report suggested that the down-regulation of FUT3 and FUT5 in tumor cells reduced the expression levels of sLe^x^ antigens, which could reduce the metastatic potential of the tumor cells process [41]. After the interaction of cells and *H. pylori* strains at 4 h, FUT1, FUT3 and FUT6 maintained a low level of expression in the study. According to the previous study, the down regulation of FUT3 could be a strategy to prevent high densities of *H. pylori* colonization in gastric cancer cell lines [41]. Several studies proposed that the increase in α1-2-fucosylation of the type 2 (Galß1-4GlcNAc-R) disaccharide led to a decrease in the synthesis of sLe^x^ [8], indicating that the increase in FUT1/2 might have an effect on the decrease in antigens catalyzed by other FUTs. Additionally, a genomic study pointed out that *H. pylori* strains with the same diseases had less variation compared to the strains with different diseases, and genetic features of *H. pylori* could be acquired to adapt to the environment [48]. Our results reveal that the expression of Fuc-related lectins binding was not obviously different between GES-1 cells infected with *H. pylori* strains from different diseases.

In conclusion, we investigated the changes in fucosylated glycans and FUTs in GES-1 cells infected with *H. pylori* strains from different sources, and our data demonstrate for the first time that the higher levels of expression of FUT1 and FUT2 were detected within 2 h post-infection. In particular, the expression of FUT2 was higher in infected GES-1 cells with a higher fold change in BC2LCN lectin specific to α1-2 linked Fuc after 4 h of infection. Alterations in carbohydrate expression in cells indicate the onset of certain diseases, fucosylation levels are generally elevated under pathological conditions such as gastritis and adenocarcinoma tissues. Epithelial fucosylated glycans govern host–microbe interactions in the digestive tract, and α1,2-fucosylated mucins in the epithelial tissues of the gastrointestinal tract act as receptors for pathogens and are involved in the activation of the immune system. Our finding indicated that the increase in α1-2 linked Fuc expression might be associated with the *H. pylori* infection of normal gastric epithelial cells. Understanding the changes of α1-2 linked Fuc in epithelial cells will lead to an understanding of the mechanisms of *H. pylori* adhesion in host gastric mucosa, thus laying the foundation for the elimination of this bacteria in the stomach.

## Figures and Tables

**Table 1:** TaqMan real-time PCR primers used in this study.

Gene	Forward Primer	Reverse Primer	Probe ^a^
*FUT1*	AGGTATAAACACACCCTCTGTGCTT	GAGTTCAGGGACAGACAGTGGTT	AAACTGGCAGGTACCGTGCTCATTGC
*FUT2*	CTCGCTACAGCTCCCTCATCTT	CGTGGGAGGTGTCAATGTTCT	TGGTCACCAGTAATGGCATGGCCTG
*FUT3*	GGGATCCCTTTTCGTCACACT	CGAACTGGTCTAAGCCTTGCA	AGGTGACCTACAGGCTCCGCTCGA
*FUT4*	AATTGGGCTCCTGCACAC	CCAGGTGCTGCGAGTTCT	TGGCCCGCTACAAGTTCTACCTGG
*FUT5*	CGCTGGATCTGGTTCAGCAT	CAGCCGTAGGGCGTGAG	CCCCCAGCAACTCCGGC
*FUT6*	GCATCCAGACGGGATCCA	ACTGCTGCGTCTTGACACCTT	CCAGGTCCCCGATCCCTCTAGCAT
*GAPDH*	GAGAAGGCTGGGGCTCAT	TGCTGATGATCTTGAGGCTG	CTCTGCTGATGCCCCCATGTTCGT

^a^ The probes were fluorescently labelled with FAM in 5′ and BHQ-1 in 3′ position. FUT: fucosyltransferase.

**Table 2:** The fold intensities of glycans expression in infected gastric epithelial (GES-1) cells with *H. pylori* strains associated with different diseases relative to uninfected cells. ^a^

Lectin	Carbohydrate Specificity	ATCC 43504-GES-1	P164-GES-1	M84-GES-1	YN4-62-GES-1	HLJ011-GES-1	HLJ030-GES-1
Lotus	αFuc	1.0	1.7	1.6	1.1	1.4	1.0
RS-Fuc	αFuc	0.5	4.2	5.1	3.3	5.8	0.9
UEA-I	αFuc	0.9	1.1	1.2	1.2	1.2	0.9
PA-IIL ^b^	Fuc, Man	1.0	1.5	1.6	1.1	2.2	1.0
AAA	Fucα1-6GlcNAc, Fucα1-3GlcNAc	0.8	1.8	1.2	2.0	1.2	1.0
AAL	Fucα1-6GlcNAc, Fucα1-3LacNAc	0.6	1.2	1.2	1.8	1.3	1.0
BC2LCN	Fucα1-2Galβ1-3GalNAc, Fucα1-2Galβ1-3GlcNAc	1.0	1.8	1.9	1.1	2.0	1.0
CNL	GalNAcβ1-4GlcNAc, GalNAcα1-3(Fucα1-2)Galβ1-4GlcNAc	1.0	1.4	1.4	1.1	1.4	1.0
MOA	Galα1-3Galβ1-4GlcNAc, Galα1-3(Fucα1-2)Gal	0.6	1.9	1.8	2.9	2.2	0.8
ACG	α2-3 Sia	0.9	2.9	2.2	2.8	3.2	0.9

^a^ Glycans color scale: red: increase (>1.2-fold) relative to control; green: decrease (>0.8-fold) relative to control; white: unchanged expression relative to control. ^b^ PA-IIL displayed affinity to fucose and mannose (Man).

## Data Availability

The data presented in this study are available on request from the corresponding author.
